# Design and Preparation of NiFe_2_O_4_@FeOOH Composite Electrocatalyst for Highly Efficient and Stable Oxygen Evolution Reaction

**DOI:** 10.3390/molecules27217438

**Published:** 2022-11-01

**Authors:** Tian-Tian Li, Bu-Yan Shi, Li-Wen Jiang, Jin-Fan Zheng, Jian-Jun Wang

**Affiliations:** 1 State Key Laboratory of Crystal Materials, Department of Neurology, Qilu Hospital, Shandong University, Jinan 250100, China; 2 Shenzhen Research Institute of Shandong University, Shenzhen 518057, China

**Keywords:** oxygen evolution reaction, electrocatalysis, NiFe_2_O_4_, thiourea-assisted synthesis, composite structure

## Abstract

Rational design and constructing earth-abundant electrocatalysts for efficient electrocatalytic water splitting is a crucial challenge. Herein, we report a simple and efficient one-step electrochemical synthetic route of the NiFe_2_O_4_@FeOOH composite electrocatalyst for the oxygen evolution reaction. The unique morphology of the NiFe_2_O_4_ nanoflowers loaded on FeOOH nanosheets allows more active sites to be exposed and promote charge transfer as well as gas release, and the resulting electrode enables a current density of 10 mA cm^−2^ at a low overpotential of 255 mV with outstanding stability at a current density of 100 mA cm^−2^ for 300 h.

## 1. Introduction

Hydrogen is considered to be one of the most promising alternates to fossil fuels due to its high energy density and non-pollution during energy conversion [1,2,3]. The electrolysis of water has attracted a large amount of attention for hydrogen production as well as the efficient storage of renewable energy [4,5,6]. However, the overall efficiency of water splitting is substantially limited by the sluggish reaction kinetics at the anode, namely, the oxygen evolution reaction (OER) [7,8,9]. A number of efficient electrocatalysts have been employed to promote the reaction rate of OER, thus improving the overall efficiency of hydrogen production by water splitting [10,11]. Until now, noble metal oxides including IrO_2_ and RuO_2_ are still considered as the benchmark electrocatalysts for OER due to their excellent electrocatalytic activities [12,13,14,15]. In recent years, tremendous effort has been paid to exploring earth-abundant non-noble metal catalysts for OER to avoid the large-scale deployment of precious metals [16,17,18,19,20].

Oxides with spinel structure including Co_3_O_4_, CoFe_2_O_4_, and NiFe_2_O_4_ have exhibited great promise as a class of electrocatalysts for OER due to low toxicity, high abundance, flexible ion arrangement, and multivalence structure [21,22,23,24]. Among them, NiFe_2_O_4_ has been extensively investigated with comparable performance to IrO_2_ and RuO_2_ [25,26,27]. For instance, Qiao and co-workers proposed a facile and reliable strategy to synthesize S- NiFe_2_O_4_/NF with thiourea-assisted electrodeposition and the resulting electrode displayed intriguing catalytic properties. By constructing heterojunction with other components, enhanced electrocatalytic activity can be achieved due to the renewed or upgraded active sites [28]. Gao et al. developed a facile solvothermal approach to decorate the NiFe LDH nanosheet with NiFe_2_O_4_ nanoparticles to form a heterostructure array on Ni foam, leading to significantly enhanced mass and charge transfer efficiency as well as the active surface [29]. Similar results were also observed from the composite electrocatalyst of FeNi/NiFe_2_O_4_/NC polyhedron-assembled microboxes derived from the carbonization of bimetal MOF [30]. Recently, our group reported the highly efficient and stable NiFe_2_O_4_−xSe_x_/NiOOH electrocatalyst on stainless steel for OER and the precise selenization of NiFe_2_O_4_ significantly boosted the OER performance. Apart from enhancing the intrinsic activity, enlarging the electrocatalytically active area and magnifying the number of active sites are of comparable importance in improving the performance of target electrodes [31,32,33,34]. Additionally, the efficiency of charge and mass transfer critically dictates the electrocatalytic activity for OER [35]. All of the aforementioned aspects concurrently contribute to the improved OER performance and thus simultaneously addressing these issues is highly desirable, but challenging.

Herein, we report on an intriguing composite electrocatalyst with spinel NiFe_2_O_4_ nanoflowers loaded on FeOOH nanoplates for OER and the resulting electrode exhibited excellent OER activity and good stability at a relatively high current density. The designed composite structure endowed the electrode with the enhanced specific surface area, consequently allowing for more active sites and efficient charge transfer as well as gas release. Electrochemical experiments demonstrated that the optimized NFO NFs@FeOOH NSs/Fe electrocatalyst showed enhanced electrocatalytic activity and enabled a current density of 10 mA cm^−2^ at an overpotential of 255 mV and outstanding stability at 100 mA cm^−2^ for 300 h. The study on mechanism revealed that thiourea has an important role during the electrochemical deposition process and significantly affects the composition and crystal structure of the composite.

## 2. Results and Discussion

**Scheme 1:**
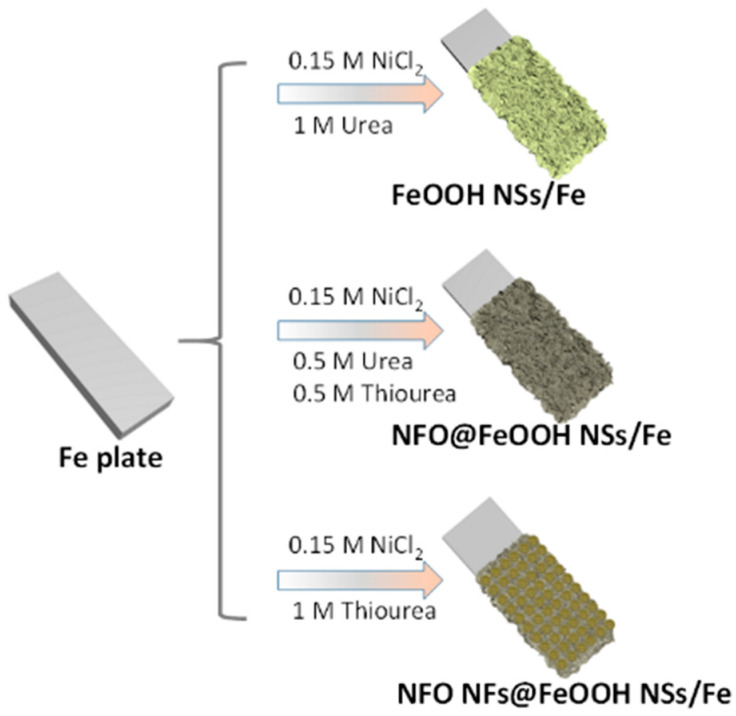
The schematic diagram of the synthesis of FeOOH NSs/Fe, NFO@FeOOH NSs/Fe, and NFO NFs@FeOOH NSs/Fe on the Fe plate via a one-step method.

The synthetic procedure of samples through a facile one-step electrodeposition method is displayed in Scheme 1. It was found that the electrolyte used is critical to the structure and morphology of the resulting samples. Typically, clean Fe plates are put into three kinds of electrolyte containing urea, thiourea, and their mixture with a ratio of 1:1 to obtain FeOOH nanosheets/Fe, NiFe_2_O_4_@FeOOH nanosheets/Fe, and NiFe_2_O_4_ nanoflowers@FeOOH nanosheets/Fe, which were denoted as FeOOH NSs/Fe, NFO@FeOOH NSs/Fe, and NFO NFs@FeOOH NSs/Fe, respectively. After drying in the air at room temperature for 12 h, the color of the samples turned into light yellow, brown yellow, and black, respectively, as shown in Figure S1.

**Figure 1:**
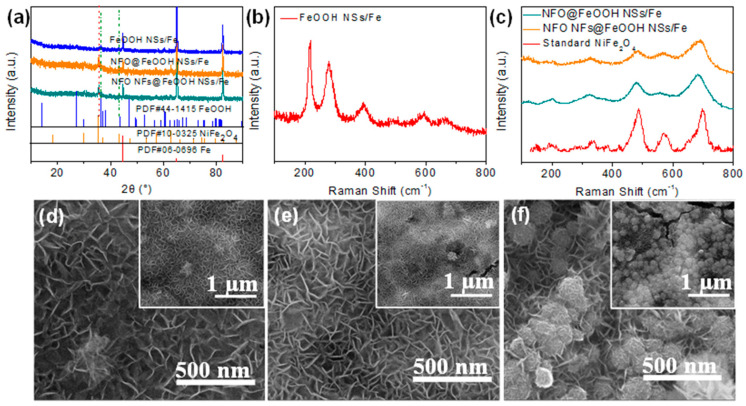
Sample characterizations. (**a**) XRD patterns of FeOOH NSs/Fe, NFO@FeOOH NSs/Fe, and NFO NFs@FeOOH NSs/Fe; (**b**) Raman spectrum of FeOOH NSs/Fe; (**c**) Raman spectrum of NFO@FeOOH NSs/Fe, NFO NFs@FeOOH NSs/Fe, and standard spinel structures NiFe_2_O_4_; the SEM images of (**d**) FeOOH NSs/Fe; (**e**) NFO@FeOOH NSs/Fe, and (**f**) NFO NFs@FeOOH NSs/Fe.

The crystal structure of the samples was first studied by XRD and Raman. In Figure 1a, stronger peaks at 44.67°, 65.02° and 82.33° were observed for all of the samples due to the (110), (200), and (211) facets of the substrate Fe plates (JCPDS No. 06-0696), respectively. In general, two sets of peaks can be observed (Figure 1a). One set of peaks at 18.39°, 30.29°, 35.69°, 37.31°, 43.36°, 47.50°, 53.80°, 57.36°, 62.91°, 66.23°, 71.48°, 74.63°, 75.56°, and 79.58° can be ascribed to the spinel NiFe_2_O_4_ structure (JCPDS No. 10-0325) and the other set of peaks at 14.11°, 27.05°, 36.29°, 36.90°, and 46.77° can be assigned to the FeOOH structure (JCPDS No. 44-1415). When pure urea was used, only the FeOOH signal was observed, suggesting that urea is profitable for the formation of FeOOH. In contrast, NiFe_2_O_4_ and FeOOH were formed when thiourea was introduced into the electrolyte. The stronger intensity of NiFe_2_O_4_ peaks than the FeOOH peaks indicates that thiourea is in favor of the formation of NiFe_2_O_4_. Additionally, the Raman spectra further consolidated the important role of thiourea on the structure and composition of the electrocatalysts. As shown in Figure 1b, the Raman peaks at ~213.6, 275.1, 379.9, and 582.7 cm^−^^1^ of the sample obtained with urea are consistent with the standard FeOOH peaks [36]. In contrast, the five Raman peaks at ~205.0, 330.0, 485.2, 560.5, and 695.1 cm^−^^1^ of the sample synthesized with thiourea (Figure 1c) can well be assigned to spinel NiFe_2_O_4_, in good agreement with the observations from XRD. No characteristic peaks of NiOOH and Ni(OH)_2_ were observed, which may be explained by the smaller solubility constant of Fe(OH)_3_ than that of Ni(OH)_2_, limiting the formation of Ni(OH)_2_ and NiOOH. Therefore, the component of the resulting composite can be readily controlled by regulating the electrolyte [25,37,38].

The morphology of the samples was studied using scanning electron microscopy (SEM) (Figure 1d–f). Clearly, FeOOH nanosheets were uniformly coated on Fe plates when the sample was prepared using urea (Figure 1d). Nanoflowers loaded on FeOOH nanosheets were observed due to the formation of NiFe_2_O_4_ assisted by thiourea (Figure 1e,f). In general, the nanoflowers are considered to be formed by the combinatorial attachment of nanoparticles or nanoplates on the existing crystal seeds due to the electrostatic interaction. Therefore, we believe that the plate-like nuclei are preferentially formed, and these plate-like nuclei are self-assembled to form flower-like crystal seeds, which are further grown to form nanoflowers. This result further proves that thiourea can regulate the morphology of the resulting samples. On the other hand, as previously reported, the deposition potential of Ni can be negatively shifted due to the depolarization effect. Clearly, the introduction of thiourea showed a critical effect on the electrodeposition process of Ni, enabling the control of Ni content in the products. Compared with the simple nanosheets, the composite structure composed of three-dimensional nanoflowers and two-dimensional nanosheets provided a larger surface area, endowing the electrode with more active sites and channels for rapid charge and mass transfer and bubble release, thus improving the catalytic performance [39,40].

**Figure 2:**
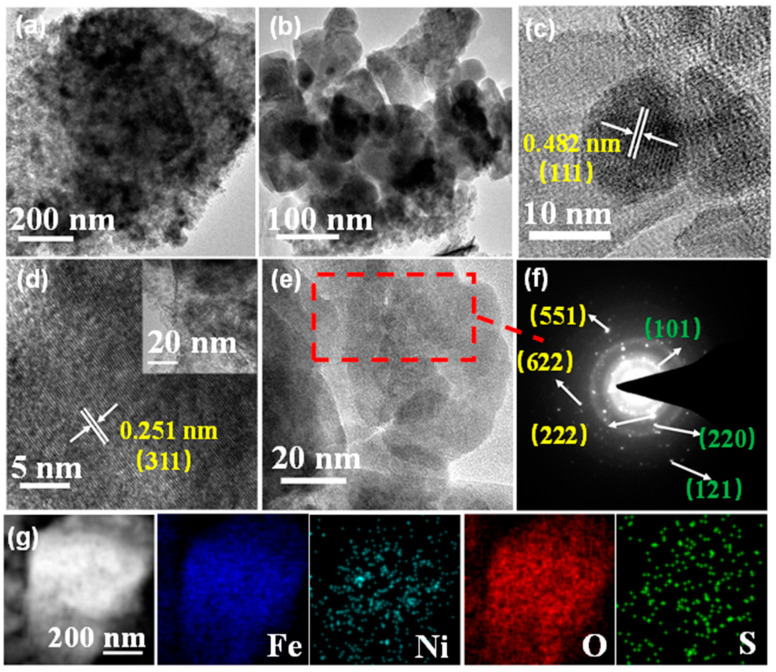
(**a**,**b**) TEM images of NFO NFs@FeOOH NSs/Fe; (**c**,**d**) HRTEM images of NFO NFs@FeOOH NSs/Fe. (**e**,**f**) The selected area electron diffraction pattern of NFO NFs@FeOOH NSs/Fe. (**g**) STEM images of NFO NFs@FeOOH NSs/Fe, and elemental mappings of Fe, O, Ni, and S.

The morphology and structure of the samples were further studied using transmission electron microscopy (TEM). As shown in Figure 2a,b, a composite structure of nanoflowers attached to nanosheets was observed and the nanoflowers were constituted of nanoparticles. The observed d-spacings of 0.482 and 0.251 nm can be ascribed to (111) and (311) planes of NiFe_2_O_4_ (JCPDS No. 10-0325), respectively, proving that the composition of nanoparticles is spinel NiFe_2_O_4_ (Figure 2c,d). Additionally, the selected area electron diffraction (SAED) pattern recorded from the region in Figure 2f indicated that the composite contained NiFe_2_O_4_ and FeOOH. The rings can be assigned to (222), (622), (551) of NiFe_2_O_4_ and (101), (220), (121) of FeOOH, respectively. The elemental mapping in Figure 2g indicated that the elements of Fe, Ni, O, and S were uniformly distributed within the NFO NFs@FeOOH NSs/Fe composite electrocatalysts. Apparently, the contents of Ni and S were significantly less than Fe and O, consistent with sulfur-doped NiFe_2_O_4_ and the FeOOH composite electrocatalyst as prepared. The results of TEM and SAED confirmed the crystal structure and components of the NFO NFs@FeOOH NSs/Fe electrocatalyst, consistent with the results of XRD and Raman. The ratio of FeOOH and NiFe_2_O_4_ in NFO NFs@FeOOH NSs/Fe was determined by TGA (Figure S2). Generally, the weight loss stage from 200 °C to 350 °C is mainly attributed to the dehydration of FeOOH to Fe_2_O_3_ [41,42]. Therefore, the contents of FeOOH and NiFe_2_O_4_ in the composite were calculated to be 4.04% and 95.96%, respectively.

**Figure 3:**
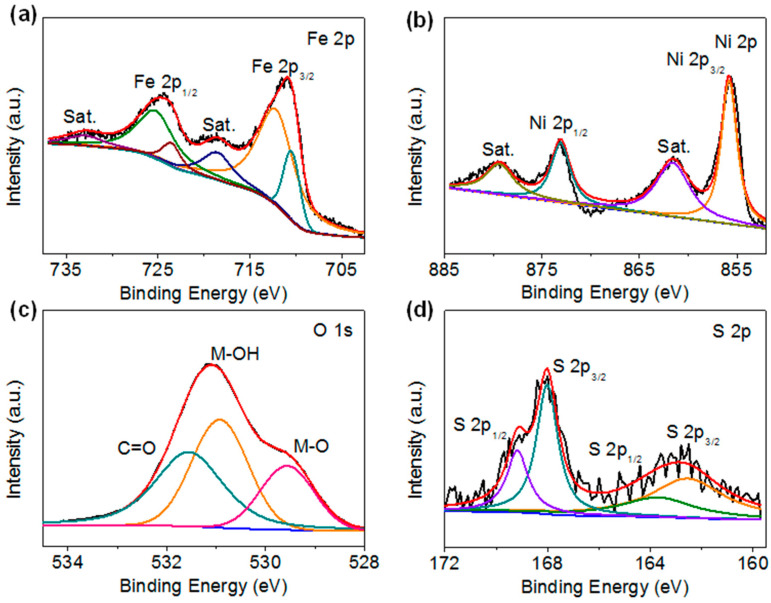
The high-resolution XPS spectra of (**a**) Fe 2p; (**b**) Ni 2p; (**c**) O 1s; and (**d**) S 2p for NFO NFs@FeOOH NSs/Fe.

The surface composition and chemical states of NFO NFs@FeOOH NSs/Fe were further investigated by XPS. The survey spectrum of NFO NFs@FeOOH NSs/Fe (Figure S3) suggests that the elements of Fe, Ni, O, and S are present, consistent with the elemental mapping analysis. In Figure 3a, the peaks at 710.9 eV (Fe^3+^ 2p3/2), 712.2 (Fe^2+^ 2p3/2), 723.7 eV (Fe^3+^ 2p1/2), and 725.8 eV (Fe^2+^ 2p1/2), along with the two satellite peaks at 718.9 eV and 732.0 eV in the Fe 2p regions, indicates the presence of Fe^3+^ and Fe^2+^, consistent with previous reports. With regard to the Ni 2p spectrum, the peaks observed at 855.7 and 873.1 eV were attributed to the spin−orbit doublets of Ni 2p3/2 and Ni 2p1/2, respectively (Figure 3b), along with two satellite peaks at 861.4 and 879.2 eV, characteristic of Ni^2+^ [27]. For the O1s spectrum displayed in Figure 3c, the two peaks at 529.5 and 530.9 eV were attributed to the M–O and M–O–H bonds, respectively, further supporting the presence of NiFe_2_O_4_ and FeOOH [43]. Notably, a peak at 531.5 eV was observed, which is likely due to the C=O bond of urea, which is caused by the decomposition of thiourea during electrodeposition [28]. The two small peaks at 162.5 and 163.7 eV corresponding to S 2p3/2 and S 2p1/2 of M–S bond, respectively, confirms the presence of S with a broad peak at about 168.0 eV due to surface oxidation (Figure 3d). Similar results were observed from the samples of FeOOH NSs/Fe and NFO@FeOOH NSs/Fe (Figures S4 and S5), further confirming the composite structure.

**Figure 4:**
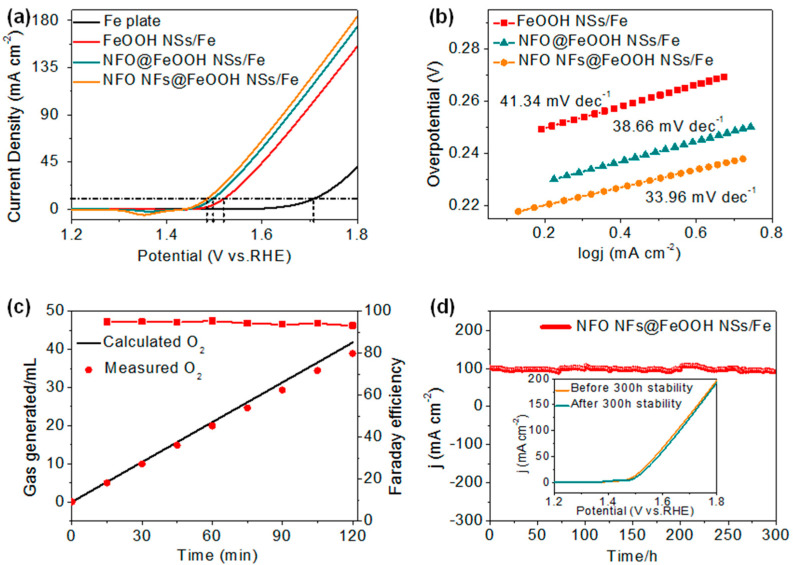
(**a**) Polarization curves of the Fe plate, FeOOH NSs/Fe, NFO@FeOOH NSs/Fe, and NFO NFs@FeOOH NSs/Fe in a 1 M KOH alkaline solution with a scan rate of 5 mV s^−^^1^ (without IR correction). (**b**) The corresponding Tafel plots; (**c**) the Faraday efficiency of NFO NFs@FeOOH NSs/Fe. (**d**) Stability of NFO NFs@FeOOH NSs/Fe at 100 mA cm^−^^2^.

To study the electrocatalytic performance of the as-prepared electrodes, a series of electrochemical characterization were carried out in 1 M KOH alkaline electrolyte. Figure 4a displays the linear sweep voltammetry (LSV) polarization curves recorded at 5 mV s^−^^1^. Clearly, the NFO NFs@FeOOH NSs/Fe composite electrocatalysts showed a significant enhanced OER performance compared with the control samples. Notably, the reduction peak between 1.3 and 1.4V(vs.RHE) is likely due to the transformation from Ni^3+^ to Ni^2+^ [44,45]. To deliver a current density of 10 mA cm^−^^2^, an overpotential of 255 mV is required for the NFO NFs@FeOOH NSs/Fe electrode, lower than FeOOH NSs/Fe (290 mV) and NFO@FeOOH NSs/Fe (267 mV). The improved OER activity of NFO NFs@FeOOH NSs/Fe was further confirmed by the Tafel plot obtained from the LSV curves according to the Tafel equation. The Tafel slope of NFO NFs@FeOOH NSs/Fe catalysts was 33.96 mV dec^−^^1^, which is lower than that of the FeOOH NSs/Fe (41.34 mV dec^−^^1^) and NFO@FeOOH NSs/Fe (38.66 mV dec^−^^1^), further indicating an improved reaction kinetics for OER [46]. The superior OER performance of NFO NFs@FeOOH NSs/Fe was even better than those of many reported promising non-precious metal oxide catalysts (Figure S10 and Table S2). The Faraday efficiency was calculated by measuring the amount of O_2_ generated using gas chromatography. In Figure 4c, a Faraday efficiency of about 95% was achieved for NFO NFs@FeOOH NSs/Fe (the calculation formula is shown in the Supplementary Materials S1), illustrating that the current truly originated from the OER process [47]. In addition, the long-term stability is critical for the practical application of OER electrocatalysts. The chronoamperometry tests indicates that NFO NFs@FeOOH NSs/Fe exhibited good stability with negligible loss for 300 h at a large current density of 100 mA cm^−^^2^. After the stability test, the catalyst displayed a comparable LSV curve to the one initially recorded (the inset of Figure 4d), implying that the excellent activity remains at a high current density in alkaline solution. The morphology of nanoflowers loaded on FeOOH nanosheets were well maintained during the stability tests at 80 h and 200 h (Figures S6 and S7), demonstrating the good structural stability of NFO NFs@FeOOH NSs/Fe.

**Figure 5:**
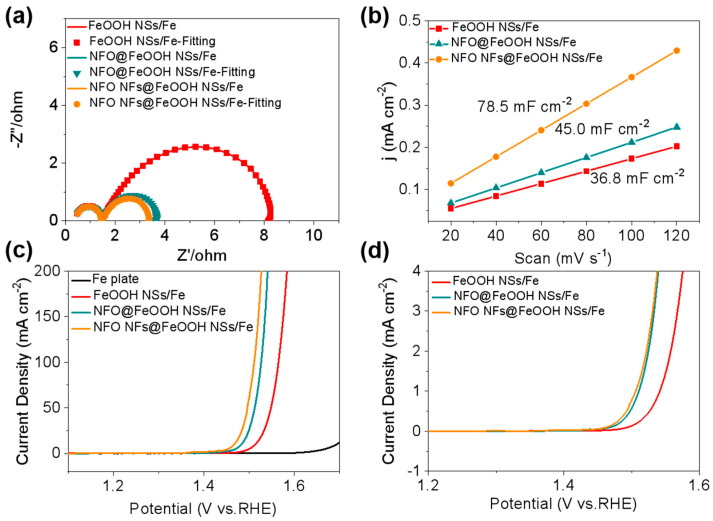
(**a**) Nyquist plots; (**b**) C_dl_ linear fitting and calculations of ECSA. (**c**) Polarization curves with 100% iR compensate, and the corresponding (**d**) ECSA normalized polarization curves.

Electrochemical impedance spectroscopy (EIS) was further performed on electrocatalysts to further study the OER kinetics and charge transfer process. The obtained Nyquist plots were fitted using an equivalent circuit (Figure S8) including a solution resistance (Rs) connected in series with two parallel combinations of resistors (R_f_, R_ct_) and constant-phase elements (CPE1, CPE2), where R_f_ signifies the charge transfer at the substrate/catalyst junction and Rct stands for the charge transfer process at the interface of the catalyst/electrolyte [48,49]. The fitting values of R_s_, R_f_, and R_ct_ are shown in Table S1. As shown in Figure 5a, all three samples showed a similar low value (~1.0 ohm) of R_f_, suggesting that self-supported electrocatalysts synthesized by the one-step deposition methods ensured tight contact of the catalyst and substrate, and promoted the charge transfer process at the interface of the catalyst/electrolyte. In contrast, the Rct of NFO NFs@FeOOH NSs/Fe (1.95 ohm) was significantly lower than FeOOH NSs/Fe (7.05 ohm) and NFO@FeOOH NSs/Fe (2.25 ohm), suggesting a faster charge transfer rate at the catalyst/electrolyte interface. The results of EIS demonstrated that the unique composite structure of the NFO NFs@FeOOH NSs/Fe electrocatalyst is conducive to reducing the charge transfer resistance at the interface, thus accelerating the charge transfer rate and improving the activity of OER. To better evaluate the intrinsic electrocatalytic activity of NFO NFs@FeOOH NSs/Fe, their electrochemical double-layer capacitance (C_dl_) was measured to evaluate the electrochemical active surface area (ECSA) (Figure S9) [50]. In Figure 5b, NFO NFs@FeOOH NSs/Fe exhibited a C_dl_ of 78.5 mF cm^−^^2^, which was much higher than that of NFS@FeOOH NSs/Fe (45.0 mF cm^−^^2^), and twice that of FeOOH NSs/Fe (36.8 mF cm^−^^2^) (the calculation formula is shown in the Supplementary Materials S2). The result of ECSA indicates that the composite electrocatalysts possesses a bigger electrochemical active surface area and consequently provides more active sites. We measured the linear sweep voltammetry (LSV) curves under automatic iR correction, as shown in Figure 5c, and NFO NFs@FeOOH NSs/Fe still displayed better OER performance than FeOOH NSs/Fe and NFO@FeOOH NSs/Fe after iR correction. Then, the iR-corrected LSV curves were normalized by ECSA to further evaluate the intrinsic activity of the samples, as displayed in Figure 5d. Notably, the LSV curve of NFO NFs@FeOOH NSs/Fe was still significantly improved compared with FeOOH NSs/Fe, but very close to NFO @FeOOH NSs/Fe after the normalization, implying that the spinel NiFe_2_O_4_ enhances the intrinsic OER activity while the unique nanoflower morphology merely exposes more ECSA without obvious effects on the intrinsic activity [51]. This further confirms that the amplified surface area of NFO NFs@FeOOH NSs/Fe composite electrocatalysts has an essential role in the improvement of OER performance, which provides more active sites, shortens the transport pathway, and accelerates OER kinetics [19,52,53]. From the EIS and ECSA results, it is clear that the unique composite structure of the NFO NFs@FeOOH NSs/Fe electrocatalyst is conducive to reducing the charge transfer resistance, thus accelerating the charge transfer rate. Moreover, compared with FeOOH NSs/Fe and NFO@FeOOH NSs/Fe nanosheet structures, NFO NFs@FeOOH NSs/Fe with a unique composite structure has a larger specific surface area, increasing the number of active sites and the contact area between the electrode and the electrolyte, which is beneficial to improving the OER performance. Additionally, the doped S can further improve the intrinsic conductivity of NFO NFs@FeOOH NSs/Fe [28]. The synergistic effect between Ni and Fe also has an essential role in improving the performance of NFO NFs@FeOOH NSs/Fe, which can regulate the electronic structure and optimize the adsorption energy of the intermediate during the OER process [54,55,56].

**Figure 6:**
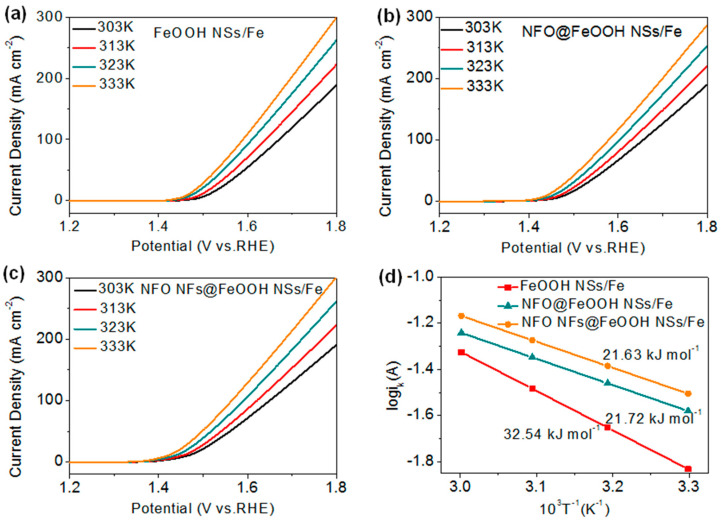
Polarization curves of (**a**) FeOOH NSs/Fe; (**b**) NFO@FeOOH NSs/Fe; and (**c**) NFO NFs@FeOOH NSs/Fe recorded at 5 mV s^−^^1^ at 303 K, 313 K, 323 K, and 333 K, respectively. (**d**) Arrhenius plots.

To understand the OER activation energy (Ea) of the prepared samples, LSV curves at varying temperatures were measured to evaluate the kinetic barriers of the corresponding OER process. In general, the reaction rate is gradually accelerated when the temperature increases (Figure 6a–c). Ea of different catalysts was calculated according to the Arrhenius relationship [57]. As shown in Figure 6d, Ea of NFO NFs@FeOOH NSs/Fe was 21.63 kJ mol^−^^1^, much lower than FeOOH NSs/Fe (32.54 kJ mol^−^^1^) and comparable to NFO@FeOOH NSs/Fe (21.72 kJ mol^−^^1^), further confirming the superiority of the unique composite structure.

According to the above analyses, the enhanced performance of NFO NFs@FeOOH NSs/Fe catalysts can be ascribed to the following aspects. First, the Tafel slope of NFO NFs@FeOOH NSs/Fe was lower than that of NFO@FeOOH NSs/Fe and FeOOH NSs/Fe, indicating that NFO NFs@FeOOH NSs/Fe possesses faster OER reaction kinetics. Second, the NFO NFs@FeOOH NSs/Fe unique composite catalysts with nanoflowers loaded on nanosheets allowed for a larger active area with more active sites, which is also an important factor for the excellent OER performance of NFO NFs@FeOOH NSs/Fe. In addition, the NFO NFs@FeOOH NSs/Fe composite electrocatalyst possessed lower charge transfer resistance, which accelerates the charge transfer and improves the electrical conductivity. Finally, in light of the Arrhenius relationship, the lower activation energy barrier value of NFO NFs@FeOOH NSs/Fe confirmed the higher intrinsic catalytic activity. Based on the experimental results and literature reports, the unique composite structure of NFO NFs@FeOOH NSs/Fe can be attributed to the following two reasons. According to the experimental results, it can be seen that urea and thiourea have a great influence on the morphology of the catalysts. A large number of studies have reported that thiourea can be decomposed into sulfur ions and urea during the electrodeposition process, which plays a regulatory role in the growth of nanocrystals. Therefore, the effect of thiourea is one of the important reasons for the formation of the unique composite structure of nanoflowers loaded on nanosheets. Additionally, the synergistic effect of Fe and Ni plays an important role in the regulation of surface morphology, which can expose more active sites, accelerate charge transfer, and improve electrical conductivity.

## 3. Materials and Methods

### 3.1. Chemicals and Materials

Nickel chloride (NiCl_2_·6H_2_O) was obtained from Macklin (Shanghai, China) and urea (CH_4_N_2_O) and potassium hydroxide (KOH) were obtained from the National Reagent Company. The commercial Fe plate (Fe content: 99.9%, thickness: 0.3 mm) was purchased from Guantai Metal Materials Co. Ltd. (Shenzhen, China). Deionized water (18.2 MΩ cm) was utilized throughout all of the experiments. All of the chemicals were analytical grade (AR) and used without additional purification.

### 3.2. Synthesis of FeOOH NSs/Fe, NFO@FeOOH NSs/Fe, and NFO NFs@FeOOH NSs/Fe Catalysts

Fe plates (3 cm × 1 cm) were used as the substrate, which were cleaned using acetone and ethyl alcohol successively for 15 min, and finally vacuum drying at 50 °C. NiFe_2_O_4_ nanoflowers@FeOOH nanosheets/Fe was prepared via thiourea-assisted electrodeposition [28,58]. In a typical procedure, 0.05 M NiCl_2_ and 1 M thiourea were mixed in 100 mL of deionized water to prepare the electrolyte solution. The electrodeposition was performed in a three-electrode cell with a CHI660E electrochemical workstation. The cleaned Fe plate, carbon rod, and Ag/AgCl electrode were utilized as the working electrode, counter electrode, and reference electrode, respectively. The deposition started at −0.9 V for 6 s and then shifted to 0.1 V for 24 s, and repeated 20 times. After electrodeposition, the sample was cleaned with deionized water and ethyl alcohol several times and then naturally dried in air. For the comparative study, the FeOOH nanosheets/Fe samples were fabricated with the electrolyte containing 1 M urea, and NiFe_2_O_4_ @FeOOH nanosheets/Fe samples were fabricated with the electrolyte containing 0.5 M urea and 0.5 M thiourea. Other steps were similar to the procedure described above. The addition of Ni^2+^ can regulate the electrodeposition process to avoid the formation of Fe_3_O_4_.

### 3.3. Material Characterization

The X-ray diffraction (XRD) patterns of samples were recorded on a SmartLab SE Advanced X-ray diffractometer (Smartlab3KW) with Cu Kα radiation (λ = 1.5418 Å). The X-ray photoelectron spectroscopy (XPS) spectrum was collected by ESCALAB 250. All of the XPS spectra were calibrated using C 1s of 284.6 eV. The Raman spectra (HORIBA LabRAM HR Evolution) were obtained using an excitation laser at 532 nm with a laser power of 2 mW. The surface morphologies of the samples were studied with a scanning electron microscope (SEM, Zeiss EVO18). Transmission electron microscopy (TEM), high resolution TEM (HRTEM, Talos F200x), and selected area electron diffraction (SAED) were also used to characterize the microstructures of the samples.

### 3.4. Electrochemical Characterizations

The electrocatalytic properties of the NFO NFs@FeOOH NSs/Fe electrocatalysts were studied in a three-electrode cell with a 1 M KOH solution as the electrolyte. A platinum plate and Ag/AgCl (3.3 M KCl) were utilized as the counter electrode and reference electrode, respectively. The as-prepared electrodes were utilized as the working electrode. The potentials used in this study were converted to ERHE from E_Ag/AgCl_ according to the formula E_RHE_ = E_Ag/AgCl_ + 0.197 + 0.05916 × pH. The polarization curves were tested at 5 mV/s. The iR compensation with a value of 100% × Ru was conducted by the automatic current interrupt method on the working station (CHI660E). Chronopotentiometry measurements were performed at 1.66 V vs. RHE in 1.0 M KOH to evaluate the stability. The Faradaic efficiency was calculated by O_2_ generation at 1.23 V vs. RHE in 1 M KOH, which was measured by gas chromatography. For comparison, the electrocatalytic activities of the Fe plate, FeOOH NSs/Fe, and NFO@FeOOH NSs/Fe samples were also measured under similar conditions. All of the experiments were carried out at ambient temperature (~300 K) and the test area for all samples was 1 cm × 1 cm.

## 4. Conclusions

A facile one-step electrodeposition method was developed to synthesize NiFe_2_O_4_/FeOOH composite electrocatalysts for efficient OER. The morphology and component of the resulting electrocatalysts can be readily controlled by changing the ratio of thiourea and urea in the electrolyte. The as-prepared electrocatalysts were studied and confirmed with XRD, SEM, TEM, and XPS. Electrochemical measurements indicate that the designed NFO NFs@FeOOH NSs/Fe composite electrocatalysts delivered a greatly improved catalytic performance for OER with a low overpotential of 255 mV to achieve a current density of 10 mA cm^−2^. Importantly, the resulting electrode exhibited a high stability for OER, allowing for at least 300 h at 100 mA cm^−2^ with negligible decay. The improved performance can be ascribed to the unique composite structure that endowed the electrode with the enhanced conductivity and specific surface area, and was consequently beneficial to electron transmission and the exposure of more active sites. This synthetic approach allows for the facile fabrication of composite OER electrocatalysts with a large surface area and good conductivity and may be extended to other potential electrodes for commercial applications.

## Data Availability

Not applicable.

## Supplementary Materials

The following supporting information can be downloaded at: https://www.mdpi.com/article/10.3390/molecules27217438/s1, Figure S1: The photographs of the FeOOH NSs/Fe, NFO@FeOOH NSs/Fe, and NFO NFs@FeOOH NSs/Fe samples after electrodeposition and dried in air; Figure S2: Thermogravimetric analysis curve of NFO NFs@FeOOH NSs/Fe; Figure S3: The XPS survey spectrum of NFO NFs@FeOOH NSs/Fe; Figure S4: (**a**) XPS survey spectrum and high-resolution XPS spectra of (**b**) Fe 2p, (**c**) Ni 2p, and (**d**) O 1s for FeOOH NSs/Fe; Figure S5: (**a**) XPS survey spectrum and high-resolution XPS spectra of (**b**) Fe 2p, (**c**) Ni 2p, and (**d**) O 1s for NFO@FeOOH NSs/Fe; Figure S6: SEM image of NFO NFs@FeOOH NSs/Fe after 80 h of stability measurement; Figure S7: SEM image of NFO NFs@FeOOH NSs/Fe after 200 h of stability measurement; Figure S8: The equivalent circuit used for fitting of Nyquist plots; Figure S9: CV curves at different scan rates. (**a**) FeOOH NSs/Fe, (**b**) NFO@FeOOH NSs/Fe, and (**c**) NFO NFs@FeOOH NSs/Fe; Figure S10: Comparison of the OER performance of NFO NFs@FeOOH NSs/Fe with the recently reported non-precious metal oxide catalysts in 1.0 M KOH electrolyte; Table S1: The obtained values of R_s_, R_f_, and R_ct_ by fitting; Table S2: Comparison of the OER performance in the 1.0 M KOH electrolyte. [27,28,41,42,48,59,60,61,62,63,64,65] are cited in the supplementary materials.

## Sample Availability

Sample of the compounds are not available.
