# Supplementary material for: Design and Preparation of NiFe2O4@FeOOH Composite Electrocatalyst for Highly Efficient and Stable Oxygen Evolution Reaction
Source: Molecules. 2022 Nov 1;27(21):7438. doi: 10.3390/molecules27217438 (PMC9654307; doi:10.3390/molecules27217438)
Supplement: Supplementary file 1 [file molecules-27-07438-s001.zip › molecules-1972412-supplementary.pdf]

Supporting Information

The $NiFe_2O_4$ nanoflowers@FeOOH nanosheets/Fe was prepared via thiourea-assisted electrodeposition according to the reactions below[1, 2].

Cathodic deposition:

$$2H_2O + 2e^- \rightarrow H_2\uparrow + 2OH^-$$

$$Ni^{2+} + 2e^- \rightarrow Ni$$

Anodic deposition:

$$Fe \rightarrow Fe^{3+} + 3e^-$$

$$Ni \rightarrow Ni^{2+} + 2e^-$$

$$2Fe^{3+} + Ni^{2+} + 8OH^- \rightarrow NiFe_2O_4\downarrow + 4H_2O$$

$$Fe^{3+} + 3OH^- \rightarrow FeOOH + H_2O$$

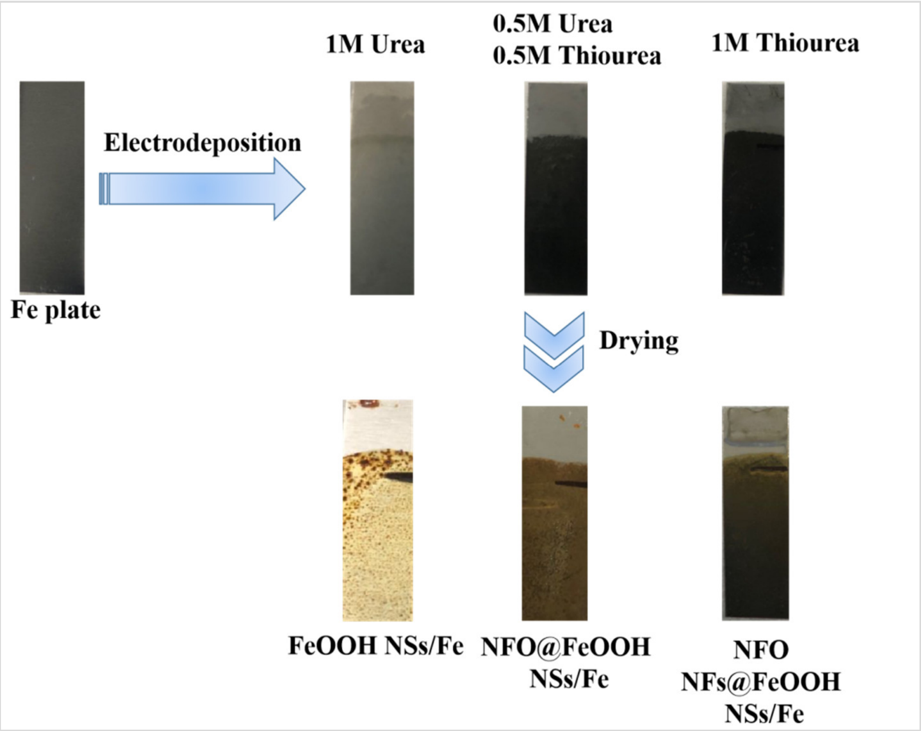

Figure S1. The photographs of FeOOH NSs/Fe, NFO@FeOOH NSs/Fe and NFO NFs@FeOOH NSs/Fe samples after electrodeposition and dried in air.

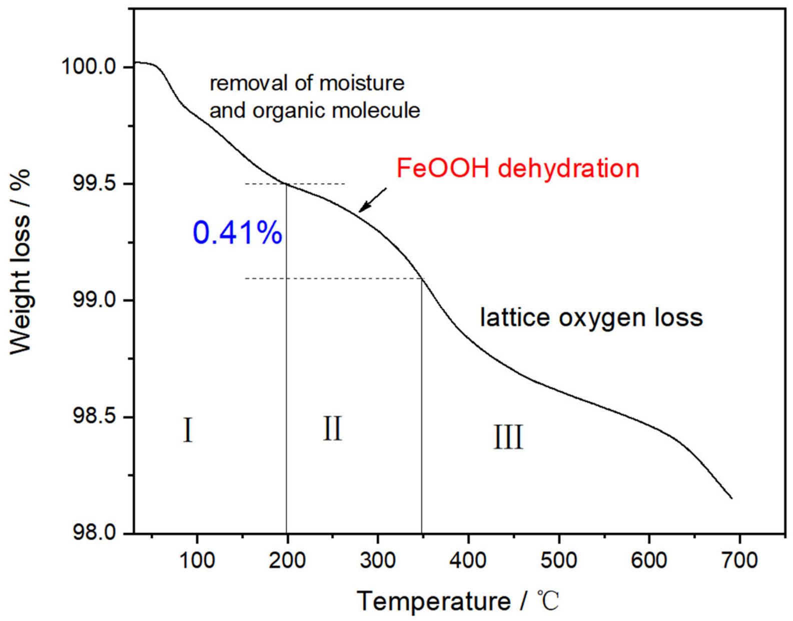

Figure S2. Thermogravimetric Analysis curve of NFO NFs@FeOOH NSs/Fe.

(I) 30 °C~200 °C: Removal of adsorbed moisture and organic molecule in the electrolyte including urea and thiourea[3].

(II) 200 °C~350 °C: Dehydration of FeOOH to Fe_2O_3[4].

(III) 350 °C~700 °C: The loss of lattice oxygen in $NiFe_2O_4$ and Fe_2O_3[5, 6].

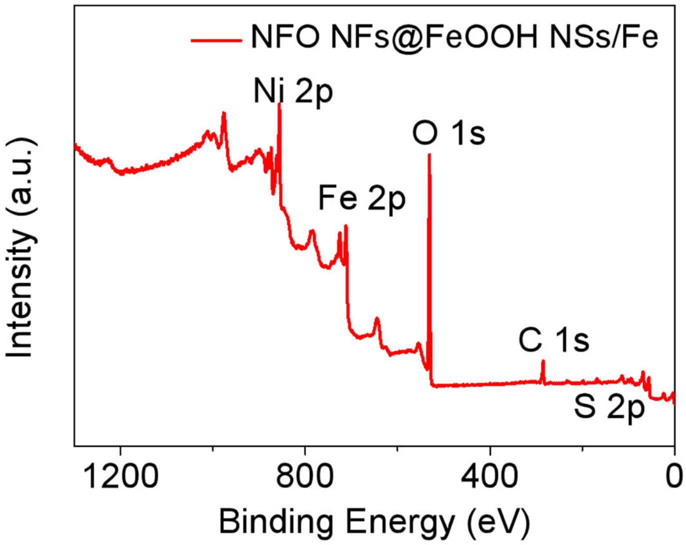

Figure S3. The XPS survey spectrum of NFO NFs@FeOOH NSs/Fe.

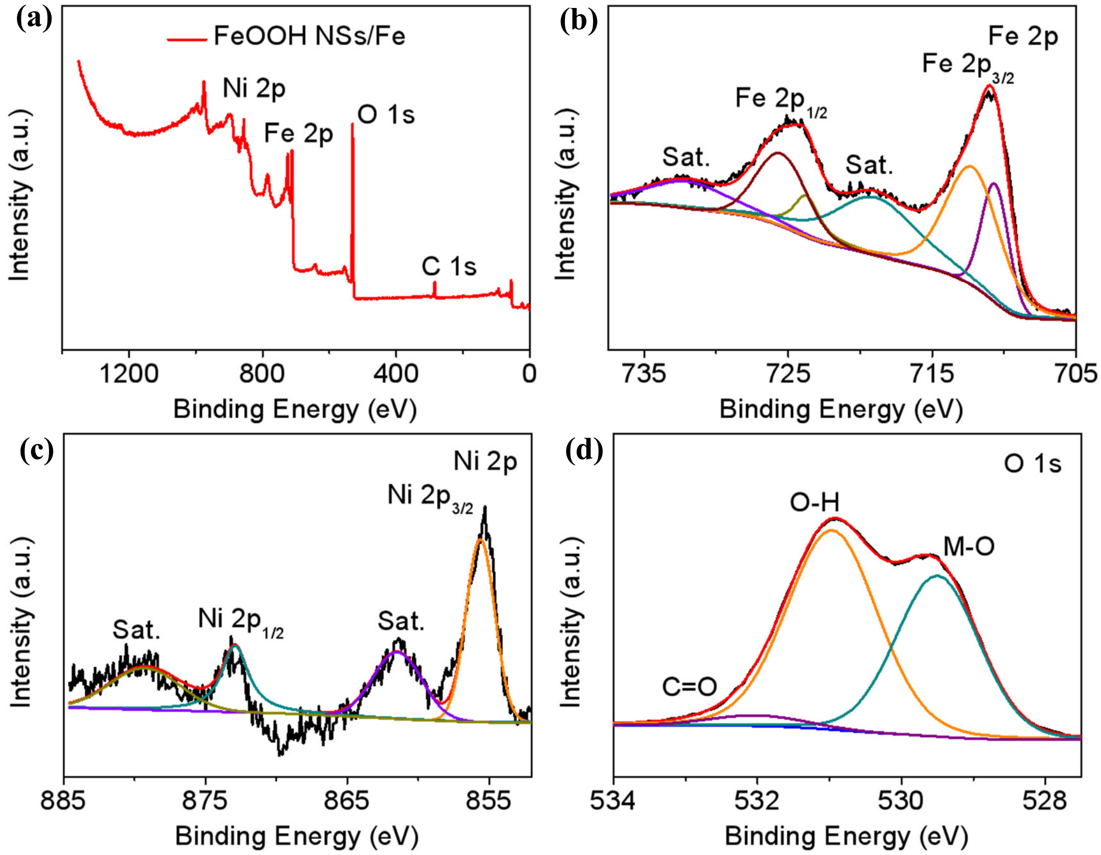

Figure S4. a) XPS survey spectrum and high-resolution XPS spectra of b) Fe 2p, c) Ni 2p and d) O 1s for FeOOH NSs/Fe.

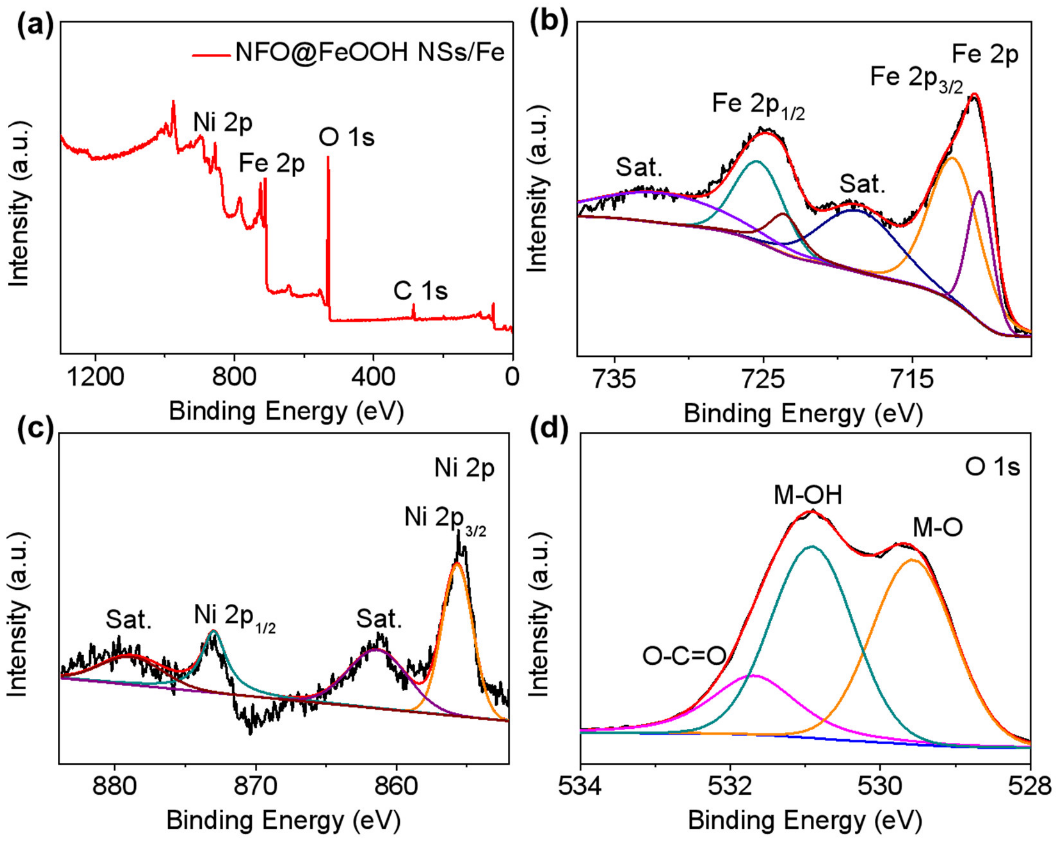

Figure S5. a) XPS survey spectrum and high-resolution XPS spectra of b) Fe 2p, c) Ni 2p and d) O 1s for NFO@FeOOH NSs/Fe.

The Faraday efficiency (FE) is calculated by the following equation:

$$FE = \frac{V_{measured}}{V_{theory}} \qquad \text{(S1)}$$

where V_{theory} represents the theoretical volume of hydrogen at a given time and $V_{measured}$ represents the measured volume of hydrogen at the same time.

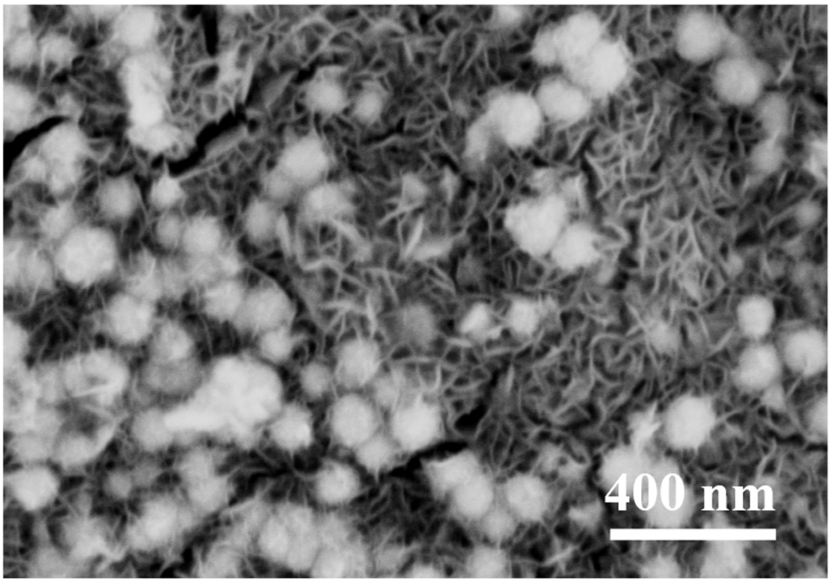

Figure S6. SEM image of NFO NFs@FeOOH NSs/Fe after 80 h of stability measurement.

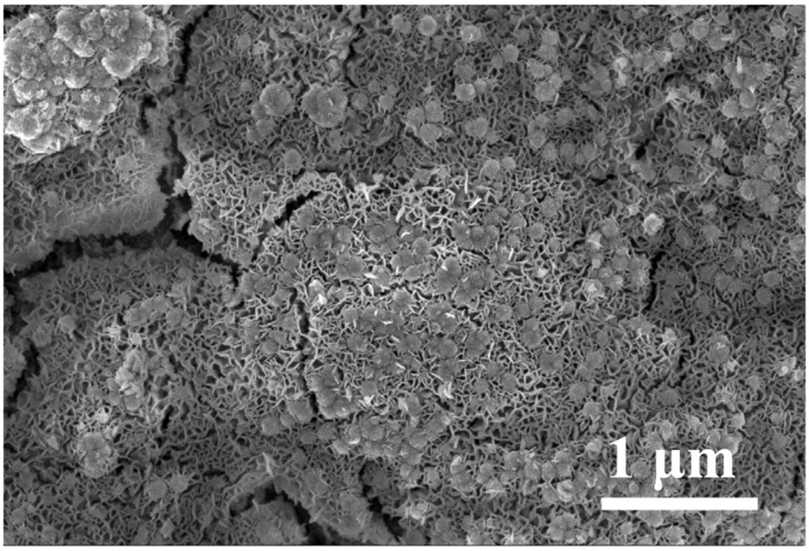

Figure S7. SEM image of NFO NFs@FeOOH NSs/Fe after 200 h of stability measurement.

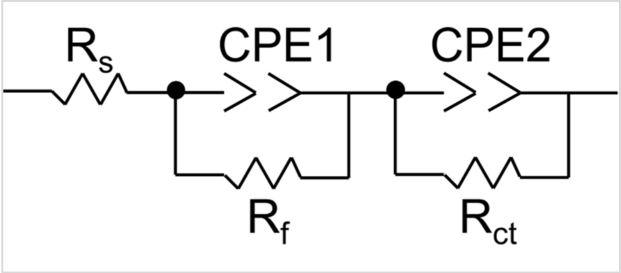

Figure S8. The equivalent circuit used for fitting of Nyquist plots.

Table S1. The obtained values of R_s, R_f, and R_{ct} by fitting.

**Sample**	R_s	R_f	R_{ct}
FeOOH NSs/Fe	0.41	1.09	7.05
NFO@FeOOH NSs/Fe	0.42	1.07	2.25
NFO NFs@FeOOH NSs/Fe	0.42	1.05	1.95

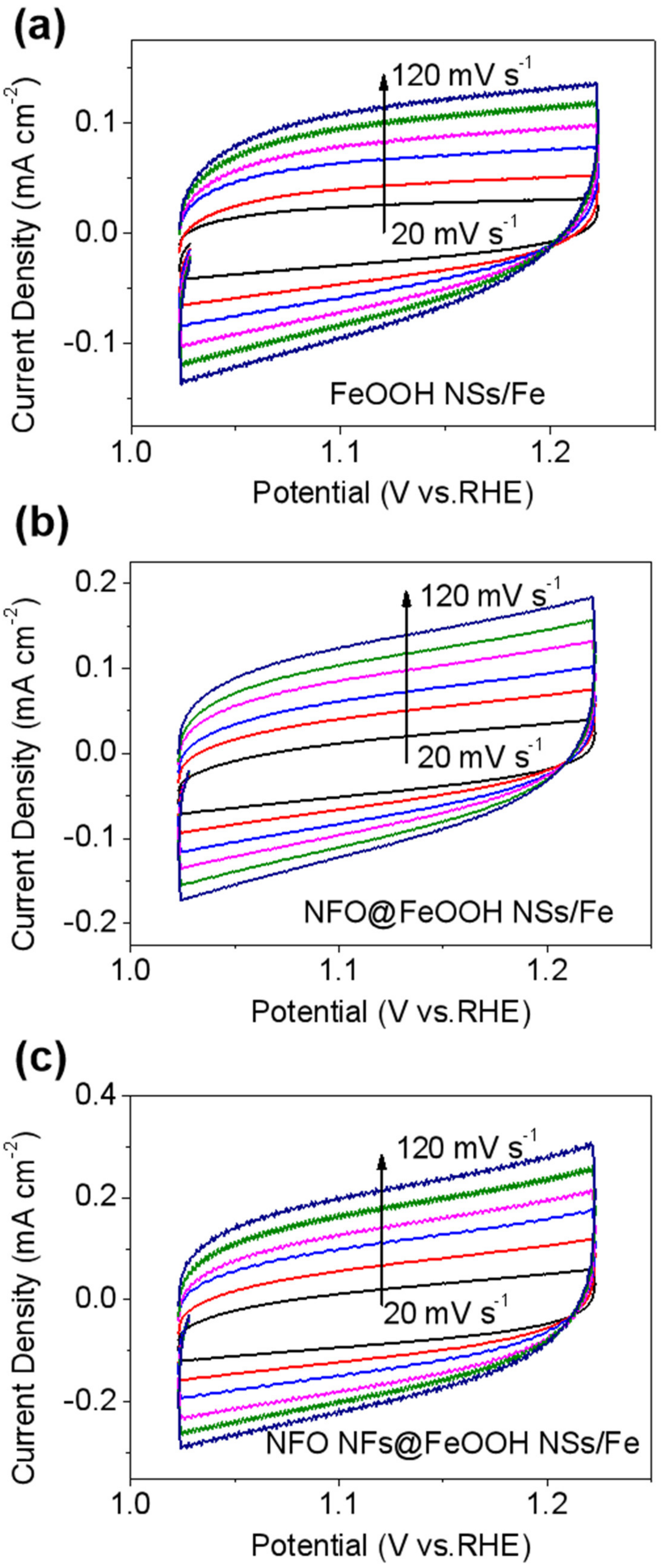

Figure S9. CV curves at different scan rates. a) FeOOH NSs/Fe, b) NFO@FeOOH NSs/Fe and c) NFO NFs@FeOOH NSs/Fe.

Double-layer capacitance (C_{dl}) was estimated based on the CV curves recorded in the non-faradaic region (1.025-1.225 V) at various scan rates. Linear slope was extracted from the plot of capacitive current densities (ΔJ, J_{anodic}-$J_{cathodic}$) at 1.125 V as a function of scan rates, where the C_{dl} was estimated as half of the linear slope.

$$ECSA = \frac{C_{dl}}{C_s} \qquad \text{(S2)}$$

where C_s is the specific capacitance of the corresponding surface-smoothed sample under the same conditions. In this work, 0.04 mF cm^{-2} was adopted as the value of C_s based on previously reported OER catalysts in an alkaline solution.

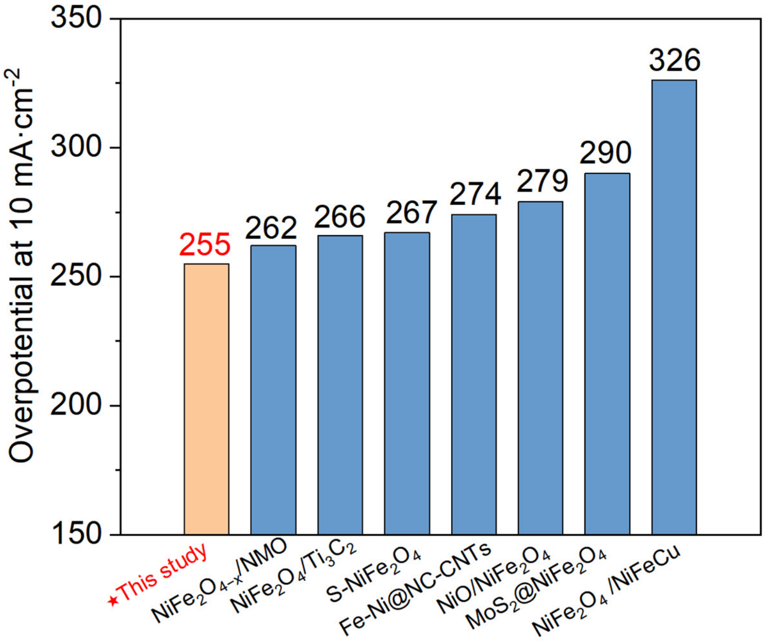

Figure S10. Comparison of OER performance of NFO NFs@FeOOH NSs/Fe with recently reported non-precious metal oxide catalysts in 1.0 M KOH electrolyte.

Table S2. Comparison of OER performance in 1.0 M KOH electrolyte.

**Electrocatalysts**	**Electrolyte**	**Electrochemical performance**	**Reference**
NFO NFs@FeOOH NSs/Fe	1 M KOH	η_{10} = 255 mV, 33.96 mV·dec^{-1}	This work
$NiFe_2O_{4-x}$/NMO	1 M KOH	η_{10} = 262 mV, 41.9 mV·dec^{-1}	Ref[7]

$NiFe_2O_4/Ti_3C_2$	1 M KOH	η_{10} = 266 mV, 73.6 mV·dec^{-1}	Ref[8]
S-$NiFe_2O_4$	0.1 M KOH	η_{10} = 267 mV, 36.7 mV·dec^{-1}	Ref[2]
Fe-Ni@NC-CNTs	1 M KOH	η_{10} = 274 mV, 45.47 mV·dec^{-1}	Ref[9]
NiO/ $NiFe_2O_4$	1 M KOH	η_{10} = 279 mV, 42.0 mV·dec^{-1}	Ref[10]
MoS_2@ $NiFe_2O_4$	1 M KOH	η_{10} = 290 mV, 69.2 mV·dec^{-1}	Ref[11]
$NiFe_2O_4$ /NiFeCu	1 M KOH	η_{10} = 326 mV, 35.9 mV·dec^{-1}	Ref[12]

References

[1] Sun, H.; Qin, D.; Huang, S.; Guo, X.; Li, D.; Luo, Y.; Meng, Q. Dye-sensitized solar cells with NiS counter electrodes electrodeposited by a potential reversal technique. *Energy Environ. Sci.* **2011**, *4*, 2630–2637.

[2] Liu, J.L.; Zhu, D.D.; Ling, T.; Vasileff, A.; Qiao, S.Z. S-NiFe2O4 ultra-small nanoparticle built nanosheets for efficient water splitting in alkaline and neutral pH. Nano Energy 2017, 40, 264–273.

[3] Safi, S.R.; Senmoto, K.; Gotoh, T.; Iizawa, T.; Nakai, S. The effect of gamma-FeOOH on enhancing arsenic adsorption from groundwater with DMAPAAQ + FeOOH gel composite. Sci. Rep. 2019, 9, 11909.

[4] Chen, X.; Zeng, Y.; Chen, Z.; Wang, S.; Xin, C.; Wang, L.; Shi, C.; Lu, L.; Zhang, C. Synthesis and Electrochemical Property of FeOOH/Graphene Oxide Composites.

Front. Chem. 2020, 8, 328.

[5] He, F.; Huang Z.; Wei, G.; Zhao, K.; Wang, G.; Kong, X.; Feng, Y.; Tan, H.; Hou, S.; Lv, Y.; Jiang, G.; Guo, Y. Biomass chemi-cal-looping gasification coupled with water/CO2-splitting using NiFe2O4 as an oxygen carrier, Energy Convers. Manag. 2019, 201, 112157.

[6] Domínguez-Arvizu, J.L.; Jiménez-Miramontes, J.A.; Salinas-Gutiérrez, J.M.; Meléndez-Zaragoza, M.J.; López-Ortiz, A.; Col-lins-Martínez, V. Study of NiFe2O4 nanoparticles optical properties by a six-flux radiation model towards the photocatalytic hydrogen production, Int. J. Hydrog. Energy 2019, 44, 12455–12462.

[7] Choi, J.; Kim, D.; Zheng, W.; Yan, B.; Li, Y.; Lee, L.Y.S.; Piao, Y. Interface engineered NiFe2O4−x/NiMoO4 nanowire arrays for electrochemical oxygen evolution. Appl. Catal. B 2021, 286, 119857.

[8] Shinde, P.V.; Mane, P.; Chakraborty, B.; Sekhar Rout, C. Spinel NiFe2O4 nanoparticles decorated 2D Ti3C2 MXene sheets for efficient water splitting: Experiments and theories, J. Colloid. Interface. Sci. 2021, 602, 232–241

[9] Zhao, X.J.; Pachfule, P.; Li, S.; Simke, J.R.J.; Schmidt, J.; Thomas, A. Bifunctional Electrocatalysts for Overall Water Splitting from an Iron/Nickel-Based Bimetallic Metal-Organic Framework/Dicyandiamide Composite, Angew. Chem.-Int. Edit. 2018, 57, 8921–8926.

[10] Zhong, H.; Gao, G.; Wang, X.; Wu, H.; Shen, S.; Zuo, W.; Cai, G.; Wei, G.; Shi, Y.; Fu, D.; et al. Ion Irradiation Inducing Oxygen Vacancy-Rich NiO/NiFe2O4 Heterostructure for Enhanced Electrocatalytic Water Splitting, Small 2021, 17,

e2103501.

[11] Karpuraranjith, M.; Chen, Y.; Wang, B.; Ramkumar, J.; Yang, D.; Srinivas, K.; Wang, W.; Zhang, W.; Manigandan, R. Hierar-chical ultrathin layered MoS2@NiFe2O4 nanohybrids as a bifunctional catalyst for highly efficient oxygen evolution and or-ganic pollutant degradation, J. Colloid. Interface. Sci. 2021, 592, 385–396.

[12] Liang, X.; Weng, W.; Gu, D.; Xiao, W. Nickel based oxide film formed in molten salts for efficient electrocatalytic oxygen evolution, J. Mater. Chem. A, 2019, 7, 10514–10522.
